# ﻿*Mitreolaquanruii* (Loganiaceae), a new species from a karst region in Guangxi, China

**DOI:** 10.3897/phytokeys.232.108986

**Published:** 2023-09-08

**Authors:** Renchuan Hu, Xiaowen Liao, Binsheng Luo, Cheng Liu, You Nong, Lei Wu

**Affiliations:** 1 Guangxi Key Laboratory of Traditional Chinese Medicine Quality Standards, Guangxi Institute of Chinese Medicine and Pharmaceutical Science, Nanning 530022, China Guangxi Institute of Chinese Medicine and Pharmaceutical Science Nanning China; 2 College of Forestry, Central South University of Forestry and Technology, Changsha 410004, China Central South University of Forestry and Technology Changsha China; 3 Lushan Botanical Garden, Jiangxi Province and Chinese Academy of Sciences, Lushan 332900, China Lushan Botanical Garden, Jiangxi Province and Chinese Academy of Sciences Lushan China; 4 CAS Key Laboratory for Plant Diversity and Biogeography of East Asia, Kunming Institute of Botany, Chinese Academy of Sciences, Kunming 650201, China Kunming Institute of Botany, Chinese Academy of Sciences Kunming China

**Keywords:** China, Loganiaceae, *Mitreola*, new taxon, taxonomy

## Abstract

*Mitreolaquanruii*, a new species from Guangxi, China, is described and illustrated in this study. It is morphologically similar to *M.liuyanii* because of the terete stems, creeping and branched at the base, the leaves which are pilose on both surfaces and the bilobed capsules with two erect horns. The new species can be distinguished from *M.liuyanii* by its taller habit, up to 20–50 cm tall, its linear leaves, 4–18 × 0.3–1 cm with acuminate apex and 8–10 pairs of lateral veins, its narrowly triangular stipules, its linear bracts, ca. 1.0 cm long and glabrous and its glabrous calyx. *Mitreolaquanruii* is temporarily assessed as data deficient (DD) according to IUCN. The habitat of *Mitreolaquanruii* is extremely fragile. Therefore, this species deserves close attention and protection.

## ﻿Introduction

*Mitreola* L. (Linnaeus 1758) is a genus of the family Loganiaceae with pantropical distribution (Chen 1995). It is generally characterised by cymose inflorescences, 5-merous flowers, cleft styles, half-inferior and bilocular ovaries and bilobed capsules with two erect or incurved horns (Leeuwenberg 1974; Wang 2018; You et al. 2020). The genus comprises about 17 species distributed in Africa, America, Asia, Oceania and the Pacific islands (Leenhouts 1962, 1972; Leeuwenberg and Vidal 1972; Leeuwenberg 1974; Li and Leeuwenberg 1996; Islas-Hernández et al. 2019; Li 2020; Liu et al. 2022). Some new species of this genus have been discovered and reported in recent years (Shan et al. 2019, 2021; You et al. 2020; Liao and Chen 2021; Liu et al. 2022). Southern and south-western China is the diversification centre of *Mitreola* and ca. 14 species have been recorded in this area so far. Of these, eleven species, most of them found in limestone areas, are endemic to the country (Fang et al. 1995; Li and Leeuwenberg 1996; Ma et al. 2010; Yu et al. 2017; Shan et al. 2019, 2021; You et al. 2020; Liao and Chen 2021; Liu et al. 2022).

Guangxi is located in the south of China. It has geomorphologically diverse landscapes, complex terrain, warm climate, abundant rain, abundant sunshine and other natural conditions, which breed rich and unique plant diversity. A total of 8,892 native plant species have been recorded in Guangxi, including 889 endemic plant species (Wei 2019). As one of the diversification centres of *Mitreola*, Guangxi has a total of seven species, amongst which five species are endemic to Guangxi and are karst obligate species (Yu et al. 2017; Wei 2019).

During our ethnobotanical field survey in Nandan County, northern Guangxi, in 2020, we collected a peculiar population of *Mitreola* with linear leaves, which was quite different from known species in the region. In the following three years, the same species was documented flowering and fruiting regularly at the same site. Careful comparison of the morphological and anatomical features of the collected taxon with other *Mitreola* species led us to believe that the taxon which we collected from Nandan differs from all the previously described species of *Mitreola*.

## ﻿Materials and methods

Several specimens were collected under evergreen broad-leaved forests in the hill region of Nandan County, Wuai Town, Tonggong Village from 2020 to 2022 and were deposited in the Herbaria CSFI, GXMI, IBK and KUN. The photographs of the plants were taken with a Panasonic LX100 camera. A detailed comparison with all other heretofore known *Mitreola* species was undertaken, including specimens deposited at CSFI, GXMG, GXMI, IBK, IBSC, HIB, KUN, PE, SYS and descriptions from botanical websites (e.g. http://www.cvh.ac.cn/, https://plants.jstor.org/). Herbarium acronyms follow Thiers (updated continuously). The morphological characters are described according to the terminology presented by Li and Leeuwenberg (1996) and the conservation status is assessed according to the IUCN Red List Categories and Criteria (IUCN 2022).

## ﻿Taxonomic treatment

### ﻿ Mitreola quanruii

Taxon classificationPlantaeGentianalesLoganiaceae

L.Wu & R.C.Hu sp. nov.

F3B643D1-83B2-5E1A-9577-15C53963B9E4

urn:lsid:ipni.org:names:77326465-1

Figs 1 , 2

#### Diagnosis.

*Mitreolaquanruii* is most similar to *M.liuyanii*, but can be distinguished from the latter by its larger plant height up to 20–50 cm tall (vs. up to 9 cm), its linear leaves, 4–18 × 0.3–1 cm (vs. leaves oblanceolate, 0.4–5.6 × 0.2–1 cm) with acuminate apex (vs. apex acute to rounded) and 8–10 lateral veins on each side of the mid-rib (vs. 4–6 pairs), its narrowly triangular stipules (vs. stipules linear), its linear bracts, ca. 1.0 cm long and glabrous (vs. bracts narrowly lanceolate, 2–3 mm long, sparsely pilose on abaxial surface) and its glabrous calyx (vs. abaxial surface of calyx sparsely pilose).

#### Type.

China. Guangxi Zhuang Autonomous Region: Nandan County, Wuai Town, Tonggong Village, growing in limestone areas, under evergreen broad-leaved forests, rare, 24°54'29.65"N, 107°21'43.83"E, 235 m a.s.l., 31 Mar 2021 (fl.), *R.C. Hu HRC210331003* (holotype: GXMI051178!, isotypes: CSFI!, IBK!, GXMI051179!).

#### Description.

Perennial herb, up to 20–50 cm tall. Stems terete, creeping, branched at the base, bearing adventitious roots; internodes 1–5 cm long, shoots sparsely pilose. Leaves opposite, papery, linear, 4–18 × 0.3–1 cm, pilose on both surfaces, base decurrent and narrowly cuneate, apex acuminate, margin ciliate, lateral veins 8–10 on each side of the mid-rib. Petioles 3–8 mm long, sparsely pilose. Stipules narrowly triangular, ca. 1 mm long, interpetiolar. Cymes terminal, 2–3-branched, many-flowered; peduncles slender, 3–8 cm long, glabrous; bracts linear, ca. 1.0 cm long, glabrous; bracteoles narrowly triangular, 1–2 mm long, glabrous; pedicels ca. 1 mm long, glabrous. Calyx lobes 5, ovate, ca. 1.3 × 1 mm, glabrous, margin membranous. Corolla urceolate, white, ca. 2 mm in diam. tube 1.3–1.7 mm long; lobes 5, ovate, 1.0–1.3 × 1.3–1.8 mm, glabrous, except for a ring of long hairs at the throat. Stamens 5, inserted near the middle of the corolla tube, glabrous, filaments ca. 0.8 mm long, anthers broadly ovate, ca. 0.3 mm long. Ovary semi-inferior, bilocular, ca. 0.5 × 1.3 mm, ovules numerous per locule; style ca. 1.2 mm long, free at base, stigma capitate. Capsules glabrous, bilobed, connate for 2/3rds of their length, with two erect horns, 1.2–3 mm long, sepals persistent at the base.

#### Phenology.

Flowering from March to April; Fruiting from May to June.

#### Distribution and habitat.

*Mitreolaquanruii* is found growing on a watery stone wall near a rivulet, under evergreen broad-leaved forests in the hill region of Nandan County, Guangxi, China.

#### Preliminary conservation status.

According to currently available data, *Mitreolaquanruii* is only found in its type locality and there are only 63 adult plants and 21 seedlings in an area of ca. 450 m^2^ (30 × 15 m). Further detailed investigation of similar habitats is needed to give a better understanding of the species’ natural distribution and abundance. *Mitreolaquanruii* is temporarily assessed as data deficient (DD) according to IUCN (IUCN 2022). At the type locality of *Mitreolaquanruii*, only a small area of native vegetation remains along the creek, surrounded mostly by plantation forests. The habitat of the *Mitreolaquanruii* is extremely fragile. Therefore, this species deserves close attention and protection.

#### Additional specimens examined.

China. Guangxi Zhuang Autonomous Region: Nandan County, Wuai Town, Tonggong Village, under evergreen broad-leaved forests, rare, 24°54'29.65"N, 107°21'43.83"E, 235 m a.s.l., 27 May 2021 (fr.), *R.C. Hu HRC210527003* (GXMI!); ibid., 28 March 2023 (fl.), *Y. Nong NY230328* (GXMI!), ibid., 23 April 2021 (fl.), *C. Liu 21CS20379* (KUN!).

#### Etymology.

We dedicate this new species of *Mitreola* to Prof. Quanru Liu for his substantial contributions to botanical research and education in China.

#### Vernacular name.

The Chinese name is proposed as xiàn yè Dù Liáng Cǎo (线叶度量草), which means that the leaves are linear.

#### Taxonomic notes.

*Mitreolaquanruii* is most similar to *M.liuyanii* because they share terete stems, creeping and branched at the base, leaves pilose on both surfaces and bilobed capsules with two erect horns. However, *M.quanruii* is well distinguished from *M.liuyanii* by its linear leaves, 4–18 × 0.3–1 cm (vs. leaves narrowly oblanceolate, 0.4–5.6 × 0.2–1 cm), with acuminate apex (vs. apex acute to rounded) and 8–10 lateral veins on each side of the mid-rib (vs. 4–6 pairs); its linear bracts (vs. bracts narrowly lanceolate). Additionally, it is morphologically similar to *M.pingtaoi* in the conspicuous, terete stems, the white corollas, the linear bracts and the capsules with two erect horns, but it differs from *M.pingtaoi* in having linear leaves, 4–18 × 0.3–1 cm (vs. leaves obovate, 2–8 × 1.2–3 cm), glabrous bracts and calices (vs. bracts and calices tomentose). Morphologically, *M.quanruii* is easily distinguishable from other species of *Mitreola* by its linear leaves. A key to the species of *Mitreola* in China is provided below.

## ﻿Discussion

Karst ecosystems are renowned for their distinct vegetation and high biodiversity, offering exceptional habitats that foster speciation and radiation (Myers et al. 2000; Biswas 2009). The genus *Mitreola* predominantly comprises limestone obligate and narrowly distributed species (Yu et al. 2017; Shan et al. 2019, 2021; Wei 2019; You et al. 2020; Liao and Chen 2021; Liu et al. 2022). Notably, recent publications have reported new species of *Mitreola* exclusively found in the limestone region of southwest China (Shan et al. 2019, 2021; You et al. 2020; Liao and Chen 2021; Liu et al. 2022). This region boasts the most extensive karst formations globally and is recognised as one of China’s three unique floristic centres, as well as a vital area for global biodiversity conservation efforts (Yu et al. 2017). In recent years, scientists have discovered and documented an increasing number of new plant species, including Annonaceae, Gesneriaceae and Magnoliaceae in this area (Hu et al. 2022; Li et al. 2022; Liu et al. 2022; Yang et al. 2023). Consequently, with further advancements in biodiversity surveys, it is expected that numerous additional species of *Mitreola* will be identified and published within the limestone regions of southwest China and northern Vietnam, potentially bringing the total count of species within this genus to thirty.

This highlights the limestone region’s ability to support rich plant diversity and endemism, while providing favourable conditions for *Mitreola*. Moreover, the unique landforms in this area have likely accelerated the diversification of *Mitreola*. Consequently, comprehensive surveys and studies on the phylogenetic evolution of *Mitreola* within the limestone areas of southwest China will yield significant scientific insights into floristic geography and the phylogeny of *Mitreola* in this particular region.

**Figure 1.**
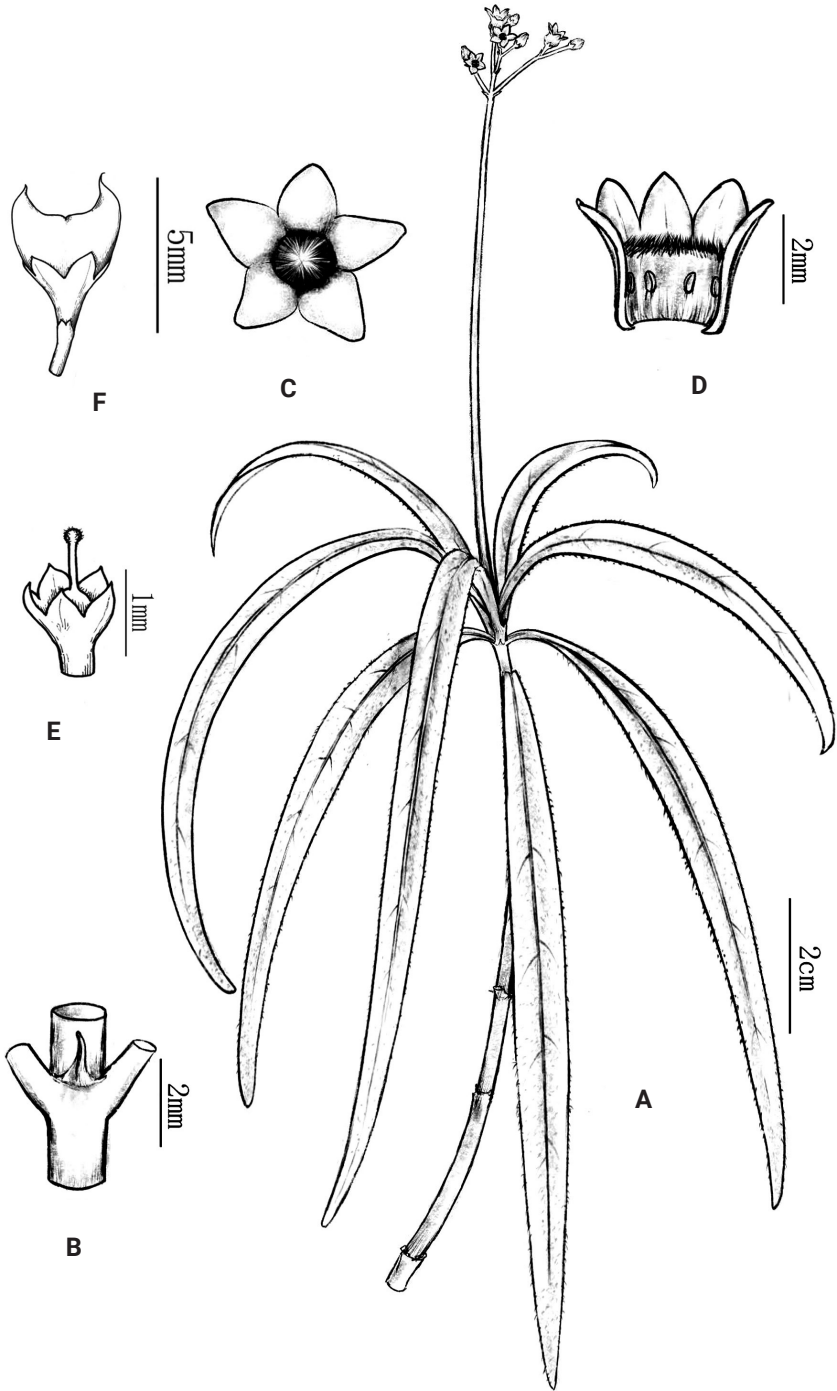
*Mitreolaquanruii* L.Wu & R.C.Hu. **A** flowering branch **B** stipule **C** top view of flower **D** longitudinally opened corolla showing the position of the stamens and the hair ring in the throat **E** ovary, calyx, style and stigma **F** lateral view of fruit.

**Figure 2.**
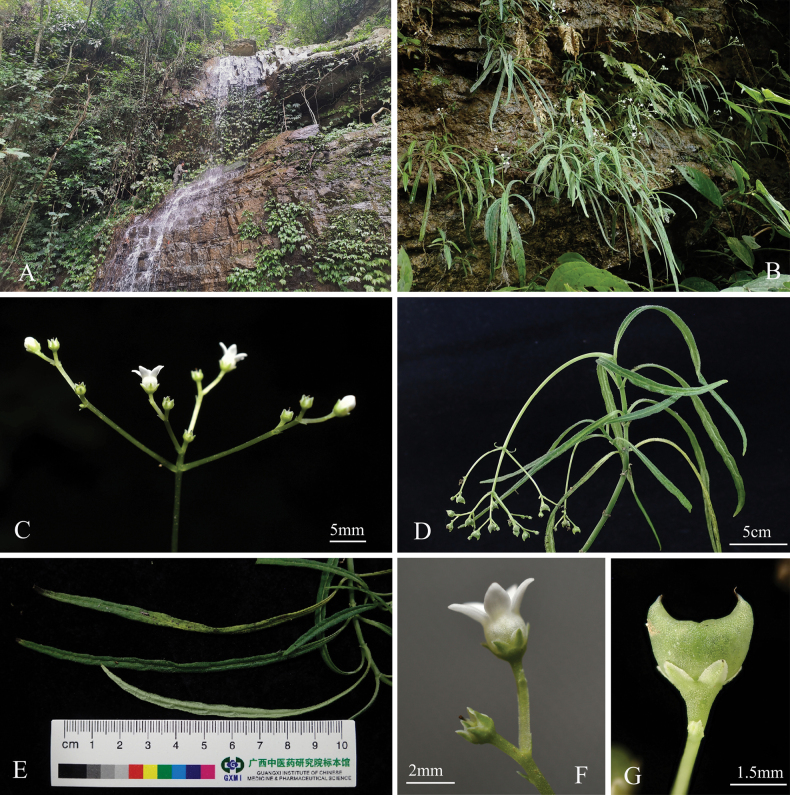
*Mitreolaquanruii* L.Wu & R.C.Hu. **A** habitat **B** habit **C** inflorescence **D** fruiting branch **E** leaves **F** lateral view of flower **G** lateral view of fruit.

### ﻿Key to species of *Mitreola* in China

**Table:** 

1	Leaves linear	***M.quanruii* L.Wu & R.C.Hu, sp. nov**
–	Leaves elliptic, ovate, lanceolate or oblanceolate	**2**
2	Stems inconspicuous; leaves in a basal rosette, sessile or subsessile	**3**
–	Stems conspicuous, creeping or erect; leaves opposite, petiolate	**5**
3	Leaves elliptic to oblong-elliptic, 3.5–7 cm long with acute apex and the veins on the lower leaf surface purple; capsule horns incurved	***M.purpureonervia* D.Fang & X.H.Lu**
–	Leaves spatulate or oblanceolate, 7–37 cm long, with obtuse or rounded apex and green veins; capsule horns erect	**4**
4	Leaf blades with 5–7 lateral veins on each side of the mid-rib; bracts narrowly triangular; stamens inserted at the middle of the corolla tube	***M.spathulifolia* D.Fang & L.S.Zhou**
–	Leaf blades with 7–10 lateral veins on each side of the mid-rib; bracts oblong; stamens inserted at the throat	***M.macrophylla* D.Fang & D.H.Qin**
5	Stems 4-angled	**6**
–	Stems terete	**9**
6	Annuals; corolla tube as long as lobes; capsule horns usually curved inwards	***M.petiolata* (J.F.Gmel.) Torr. & A.Gray**
–	Perennials; corolla tube longer than lobes; capsule horns or lobes erect	**7**
7	Corolla lobes blue; stamens inserted at the base of the corolla tube	***M.crystallina* Y.M.Shui & W.H.Chen**
–	Corolla lobes white; stamens inserted at the middle of the corolla tube	**8**
8	Leaves glabrous; stipules with glandular hairs at the margin; peduncles 1–2.5 cm long	***M.reticulata* Tirel**
–	Leaves abaxially pubescent when young; stipules glabrous at the margin; peduncles 3–7 cm long	***M.pedicellata* Benth.**
9	Stems erect; stamens inserted at the base of the corolla tube	**10**
–	Stems erect or creeping; stamens inserted at or near the middle of the corolla tube	**11**
10	Annuals; leaf blades ovate, 0.5–2 cm long, apex obtuse	***M.petiolatoides* P.T.Li**
–	Perennials; leaf blades not ovate, 1–10 cm long, apex acute	***M.liui* X.L.Du & Z.J.Mu**
11	Leaf blades obovate or oblanceolate, with 4–6 lateral veins on each side of the mid-rib	**12**
–	Leaf blades elliptic, with 7–10 lateral veins on each side of the mid-rib	**13**
12	Leaf blades narrowly oblanceolate, 0.6–7.4 × 0.2–1 cm	***M.liuyanii* C.Liu & M.Q.Han**
–	Leaves obovate, 2–8 × 1.2–3 cm	***M.pingtaoi* D.Fang & D.H.Qin**
13	Plant 3.0–8.5 cm tall; leaf blades bullate; calyxes purplish-red; corollas light purple	***M.bullata* Y.S.Chen & J.J.Liao**
–	Plant 8–60 cm tall; leaf blades smooth; calyxes green; corollas white	**14**
14	Leaves 4–10.2 × 1.8–3.8 cm, apex acute; capsules with two erect horns	***M.yangchunensis* Q.X.Ma, H.G.Ye & F.W.Xing**
–	Leaves 3–5 × 1–1.6 cm, apex pungent; capsules with two horns curved inwards	***M.lincangensis* Z.J.Mu, Z.J.Shan & B.Pan**

## Supplementary Material

XML Treatment for Mitreola quanruii

## ﻿Additional information

### Author ORCIDs

Renchuan Hu https://orcid.org/0000-0002-0941-7203

Xiaowen Liao https://orcid.org/0000-0003-2652-2862

Cheng Liu https://orcid.org/0000-0001-7146-2542

### Data availability

All of the data that support the findings of this study are available in the main text.
